# AUS-TBI: The Australian Health Informatics Approach to Predict Outcomes and Monitor Intervention Efficacy after Moderate-to-Severe Traumatic Brain Injury

**DOI:** 10.1089/neur.2022.0002

**Published:** 2022-06-07

**Authors:** Melinda Fitzgerald, Jennie Ponsford, Natasha A. Lannin, Terence J. O'Brien, Peter Cameron, D. James Cooper, Nick Rushworth, Belinda Gabbe

**Affiliations:** ^1^ Curtin Health Innovation Research Institute, Curtin University, Nedlands, Western Australia, Australia.; ^2^ Perron Institute for Neurological and Translational Science, Nedlands, Western Australia, Australia.; ^3^ School of Psychological Sciences, Monash University, Melbourne, Victoria, Australia.; ^4^ Monash Epworth Rehabilitation Research Centre–Epworth Healthcare, Richmond, Victoria, Australia.; ^5^ Department of Neuroscience, Central Clinical School, Monash University, Melbourne, Victoria, Australia.; ^6^ Department of Epidemiology and Preventive Medicine, Monash University, Melbourne, Victoria, Australia.; ^7^ Australian and New Zealand Intensive Care Research Centre Recovery Program (ANZIC-RC), Monash University, Melbourne, Victoria, Australia.; ^8^ School of Public Health and Preventive Medicine, Monash University, Melbourne, Victoria, Australia.; ^9^ Brain Injury Australia, Sydney, New South Wales, Australia.

**Keywords:** assessment tools, biomarkers, epidemiology, human studies, traumatic brain injury

## Abstract

Predicting and optimizing outcomes after traumatic brain injury (TBI) remains a major challenge because of the breadth of injury characteristics and complexity of brain responses. AUS-TBI is a new Australian Government–funded initiative that aims to improve personalized care and treatment for children and adults who have sustained a TBI. The AUS-TBI team aims to address a number of key knowledge gaps, by designing an approach to bring together data describing psychosocial modulators, social determinants, clinical parameters, imaging data, biomarker profiles, and rehabilitation outcomes in order to assess the influence that they have on long-term outcome. Data management systems will be designed to track a broad range of suitable potential indicators and outcomes, which will be organized to facilitate secure data collection, linkage, storage, curation, management, and analysis. It is believed that these objectives are achievable because of our consortium of highly committed national and international leaders, expert committees, and partner organizations in TBI and health informatics. It is anticipated that the resulting large-scale data resource will facilitate personalization, prediction, and improvement of outcomes post-TBI.

## Introduction

### The burden of TBI: Australia and the global context

Traumatic brain injury (TBI) results from diverse mechanisms of injury including road-traffic, domestic, workplace, sport and interpersonal violence.^1^ It can be catastrophic, with a lifelong impact on the persons who experience the injuries, their families and support systems, carers, workplaces, healthcare, disability, welfare, and the criminal justice systems, and society.^2–11^ The incidence of TBI in Australia is difficult to describe accurately, particularly for mild TBI where people do not always access medical care.^1,12^ There were an estimated 275 (230–327) cases per 100,000 population in 2016.^1^ More recent estimates that extrapolate incidence of moderate-severe TBI from the Victorian State Trauma Registry to the national context are 46 cases per 100,000 population.^13^ Data from New Zealand are also informative because of similarities in population demographics and lifestyles. An estimated incidence of 790 TBI cases per 100,000 person-years in New Zealand^14^ extrapolates to between 190,000 and 200,000 cases per year in Australia, of which ∼20,000 may be moderate-to-severe injuries.

TBI has consistently been the leading cause of post-injury mortality, without successful breakthroughs in treatment.^15^ Severe TBI has a high mortality rate of 30–40%, with <50% of survivors returning to full independence and productive activity.^16,17^ New cases of moderate-to-severe TBI add $2 billion in direct lifetime costs to the Australian healthcare system annually.^2^ Between 2006 and 2015, there has been no substantial change in survival or functional outcomes post-TBI in Victoria, Australia,^18^ highlighting the opportunity for better and/or more targeted treatments to reduce mortality, improve quality of life, and reduce the negative impacts on families and society.

## Discussion

### The impact of variations in injury, patient factors, environment, and care systems

The rate and degree of patients' recovery after moderate-to-severe TBI vary greatly, attributable, in part, to the complex and diverse nature of these injuries and also because of many pre-morbid clinical, psychological, and social factors. The huge heterogeneity of TBI severity motivates a precision-based approach to treatment. Yet, despite decades of empirical research, targeting treatments and predicting individual outcomes after TBI remains challenging and imprecise. With disparate disconnected data systems, we have an incomplete understanding of what it is about the person, their injury, their environment, and/or their care that moderates and/or determines the multiple outcomes that contribute to functional outcomes and quality of life. Clinical decision making in prediction and management remains inconsistent.^19,20^ Currently, no indicator or group of indicators can reliably predict treatment outcomes for an individual person with TBI, nor that person's responsiveness to therapies, to enable effective, personalized acute care and rehabilitation/follow-up for individual Australian persons with moderate-to-severe TBI.^21–23^

On a global scale, significant variations in care and access to care, as well as disparate approaches to data collection, confound our ability to interpret the effects of interventions and generalize findings to the Australian context. Australia's healthcare system has geographical challenges, with the need for retrieval and transfer of patients from remote locations. There is now an urgent need to standardize approaches to capture and link data and harmonize measures when assessing interventions across sites and contexts, to enable personalization of care and treatment for persons with TBI.

### Progress to date

#### Existing data sets do not meet the Australian need

Large-scale international consortia, gathered under the International Initiative for Traumatic Brain Injury Research (InTBIR), represent an exemplar for data-driven health informatics approaches to understanding and improving TBI care. InTBIR includes the multi-center observational comparative effectiveness research study known as the Collaborative European NeuroTrauma Effectiveness Research (CENTER-TBI) initiative, an observational cohort study focusing on the importance of systems-of-care variations. It has collected acute clinical, blood, and imaging data from 4509 TBI cases of all severity, including 1375 moderate-to-severe TBI participants.^24,25^ The Transforming Research and Clinical Knowledge in Traumatic Brain Injury (TRACK-TBI) initiative^26^ has enrolled 3000 patients with mild-to-severe TBI from major trauma centers in the United States. Mild TBI constitutes the bulk of this cohort and is the focus of published studies to date.^5,27^ The Australasian Paediatric Research in Emergency Departments International Collaborative (PREDICT) has determined optimal clinical imaging strategies in a large cohort of children with TBI,^28^ in association with national paediatric rehabilitation services.

Nonetheless, these contributions do not fully meet the needs of the Australian context. Existing large-scale data sets do not include Australian persons who experience moderate-to-severe TBI, particularly populations with disproportionate representation—for example, Aboriginal and Torres Strait Islander persons.^29^ Nor do existing data sets integrate multiple consistent outcome measures of value to persons with lived experience of the diversity of these injuries. Moreover, current predictive models using these data lack the sensitivity and specificity to personalize care at the level of a person with TBI, and trials of TBI interventions are not integrated with large-scale data collection.

#### Current predictive models lack general applicability

Existing prognostic models for moderate-to-severe TBI to assist early clinical decision making have been derived from hospital admission data, for example, from the International Mission on Prognosis and Analysis of Clinical Trials (IMPACT) and Corticosteroid Randomization After Significant Head Injury (CRASH) trials. Age, the motor score component from the Glasgow Coma Scale and pupillary reactivity, combined with specific computed tomography findings and occurrence of secondary insults (hypoxia/hypotension), provide percentage risks for poor outcome (area under curve 0.801 and 0.796 for mortality and unfavorable functional outcome at 6 months, respectively).^30^ Recent CENTER-TBI studies have validated the IMPACT and CRASH models, finding that the models identify patients at high risk for mortality or unfavorable outcome.^31^ Notably, the models were designed to predict “unfavourable outcome” with a dichotomised Glasgow Outcome Scale score, an approach that does not address all outcomes of value to the person with TBI and their families,^26^ including aspects of community participation and psychological adjustment.^21,23^

Large-scale international efforts to predict outcomes after severe TBI, including the IMPACT study, have identified the need for specific imaging parameters to add to current predictive factors.^25,32,33^ The CENTER-TBI Study validated the National Institute of Neurological Disorders and Stroke Common Data Elements for radiological findings, showing that a subset of radiological data improves outcome prediction.^34^ A longitudinal cohort studied in Victoria, Australia^35^ has identified the significant impact on outcomes of pre-injury demographic, mental health, and social factors as well as injury factors, including duration of post-traumatic amnesia, on outcomes,^23,36–38^ but this has focused mainly on road-trauma victims and has not captured the full range of injury causes. It is likely that the general applicability of current predictive models could be improved with additional data elements, assessed within the context of broader populations, including Aboriginal and Torres Strait Islander persons.

### AUS-TBI

#### A major contributor to the traumatic brain injury global knowledge commons

Funded by the Australian Federal Government Medical Research Future Fund Mission for TBI, AUS-TBI will, in the first instance, design the health informatics approach to collect and integrate nationally representative data from persons who have experienced moderate-to-severe TBI. Other approaches under the funding initiative will collect data from persons who have experienced mild TBI and cover more focused acute-care aims.^39^ We believe that these data, building on the foundations of past InTBIR studies, will make a significant contribution to the knowledge network and information commons for TBI.^40^

#### Determining data elements

**FIG. 1.**
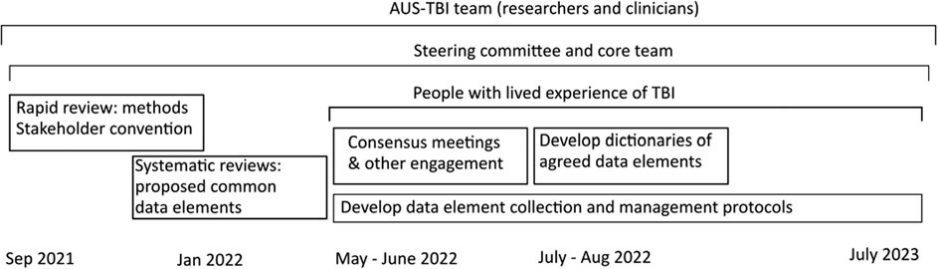
Schematic for determining data elements in TBI research, covering the entire trajectory of a person's journey. TBI, traumatic brain injury.

The AUS-TBI team encompasses researchers and clinicians expert in all areas of TBI care and data management. The team will determine the most potentially useful common data elements that can be feasibly and reliably collected from all patients in Australia who sustain these injuries. The chosen common data elements will enable harmonization with existing international data sets to maximize our capacity to generate prognostic models that are useful for persons with TBI. We will utilize an evidence-based, consensus approach to identify a broad range of suitable data elements that may be useful for predicting outcomes in Australian patients, which could also be used to evaluate the efficacy of interventions in clinical trials. The consensus approach will draw upon systematic literature searches and the views of persons with lived experience of TBI through roundtable meetings and other facilitated engagement. The data elements will include social, biological, health, clinical, intervention, and outcomes that are of value to persons with lived experience of TBI. For the first time in TBI research, the data elements considered will: cover the entire trajectory of a person's journey, from injury to reintegration back into the community; include persons from all States and Territories in Australia; encompass all demographics; and span the diversity of clinical presentations of persons with moderate-to-severe TBI, including persons with multiple other injuries and/or comorbidities (Fig. 1).

#### A framework for data collection, linkage, and management

The AUS-TBI team will also design a health informatics approach to optimally collect, link, store, manage, and protect the data, thereby securely and efficiently facilitating broad access to a nationally consistent, high-quality, harmonized, and linked data set. The resultant data, once collected, will stimulate research to develop optimized, evidence-based care for TBI through improved decision making and healthcare pathways for moderate-to-severe TBI. We plan to design an approach that links TBI data to routinely collected multi-sectoral administrative data sets and apply machine learning to develop accurate prediction models aimed at reducing TBI mortality and morbidity. It is hoped that the algorithms generated in an Australian context will be designed to assist healthcare workers in best-practice approaches to improve functional outcomes, optimise cost-effectiveness of treatment and care from a whole-of-government perspective,^41–44^ and provide the foundation for adoption to improve TBI treatment globally.

The AUS-TBI team will design the health informatics approach based on best practice, drawing from the substantial expertise of the national and international Investigator team. Consumers and stakeholders, including Aboriginal and Torres Strait Islander persons, will codesign at every stage to ensure that outcomes are of value to persons with lived experience of TBI^45^ and data collection and management is culturally safe. We will follow the FAIR data principles of Findable, Accessible, Interoperable and Reusable.^46^ Use of common data elements as outcome measures will minimize duplication and data collection burden for patients and families and allow benchmarking and comparisons of effectiveness across multiple trials. We will work with partner organizations to ensure the applicability and appropriateness of the recommended health informatics approach to maximize future translation. Given that injuries are a key driver of health inequalities for Aboriginal and Torres Strait Islander persons,^47^ a national Aboriginal and Torres Strait Islander Expert Advisory Committee will ensure that AUS-TBI identifies the optimal measures to ensure the accuracy of follow-up data in these vulnerable and often remotely located populations.

### Novelty of the approach

In a first for Australian TBI research, the acute care, rehabilitation, community, and research sectors have been brought together, including those in regional and remote Australia. In addition, this data resource will help bridge the gap between child and adult services, which is currently exacerbated by separation of data sets. We will utilize innovative linkage methods, considering privacy preserving record linkage^48^ to encode and link relevant data sets allowing for the tracking of patients across health and social services and across time. Routinely integrating multiple measures to improve the prediction of outcomes, social integration, employment, and later academic achievement^49^ will assist in the planning of services. The data resource may provide measures that can predict risk of neurodegeneration post-TBI, providing a substantial breakthrough that can inform care, as well as health and social policy. The focus of the data resource on prediction of outcomes after moderate-to-severe TBI will, by its nature, limit the choice of common data elements to those that achieve this fundamental aim of the project. As a result, some potential avenues of investigation may not be included, such as exploratory biomarker analyses. Common data elements will be limited to those that are feasible to collect from all participating institutions, which may further limit scope.

#### Expected benefits

More accurate and individual patient-specific prognostication is critical for counseling persons with TBI and their families, and we see AUS-TBI as a key step toward meeting that need. Identifying specific pathophysiological features associated with poor outcomes after TBI will inform the design of new treatments, such as novel pharmacotherapies and rehabilitation therapies. The goal is for clinicians to use and build on the data to determine the most effective treatments on an individual basis, allowing for more targeted therapy. The approach will allow for the accurate tracking of treatment responses through evidence-based, consensus-derived biomarkers, thereby aiding interpretation and increasing the success of future clinical trials. The approach will consider the integration of multi-site trials of interventions into the data collection and health informatics system, to support streamlined, efficient, and cost-effective clinical trials. Importantly, this will include targeted activities, including Aboriginal and Torres Strait Islander persons based on accepted codesign and culturally sensitive methodologies and, in doing so, address a key limitation of international data sets. The project will create the blueprint for a data resource that will facilitate world-leading interdisciplinary TBI research that addresses the needs of consumers. The data resource will create an investment case and aid policy makers in resource allocation to respond to moderate and severe TBI effectively.

## Supplementary Material

Supplemental data

## Supplementary Materials

The AUS-TBI Investigators

## Abbreviations Used

CRASH: Corticosteroid Randomization After Significant Head Injury
IMPACT: International Mission on Prognosis and Analysis of Clinical Trials
InTBIR: International Initiative for Traumatic Brain Injury Research
TBI: traumatic brain injury

## References

[1] GBD 2016 Traumatic Brain Injury and Spinal Cord Injury Collaborators. (2019). Global, regional, and national burden of traumatic brain injury and spinal cord injury, 1990–2016: a systematic analysis for the Global Burden of Disease Study 2016. Lancet Neurol. 18, 56–87. 30497965 10.1016/S1474-4422(18)30415-0 PMC6291456

[2] Access Economics Pty Ltd; Victorian Neurotrauma Initiative. (2009). The Economic Cost of Spinal Cord Injury and Traumatic Brain Injury in Australia. Geelong, Victoria, Australia: Victorian Neurotrauma Initiative.

[3] Graff, H.J., Siersma, V., Moller, A., Egerod, I., and Rytter, H.M. (2020). Five-year trends in marital stability, academic achievement, and socioeconomic indicators after concussion: a National Register study. J. Head Trauma Rehabil. 35, E86–E94. 31246879 10.1097/HTR.0000000000000501

[4] Mikolic, A., Polinder, S., Steyerberg, E.W., Retel Helmrich, I.R.A., Giacino, J.T., Maas, A.I.R., van der Naalt, J., Voormolen, D.C., von Steinbuchel, N., Wilson, L., Lingsma, H.F., and van Klaveren, D.; CENTER-TBI Participants and Investigators. (2021). Prediction of global functional outcome and post-concussive symptoms after mild traumatic brain injury: external validation of prognostic models in the Collaborative European NeuroTrauma Effectiveness Research in Traumatic Brain Injury (CENTER-TBI) Study. J. Neurotrauma 38, 196–209. 32977737 10.1089/neu.2020.7074

[5] Nelson, L.D., Temkin, N.R., Dikmen, S., Barber, J., Giacino, J.T., Yuh, E., Levin, H.S., McCrea, M.A., Stein, M.B., Mukherjee, P., Okonkwo, D.O., Diaz-Arrastia, R., Manley, G.T., Adeoye, O., Badjatia, N., Boase, K., Bodien, Y., Bullock, M.R., Chesnut, R., Corrigan, J.D., Crawford, K., Duhaime, A.C., Ellenbogen, R., Feeser, V.R., Ferguson, A., Foreman, B., Gardner, R., Gaudette, E., Gonzalez, L., Gopinath, S., Gullapalli, R., Hemphill, J.C., Hotz, G., Jain, S., Korley, F., Kramer, J., Kreitzer, N., Lindsell, C., Machamer, J., Madden, C., Martin, A., McAllister, T., Merchant, R., Noel, F., Palacios, E., Perl, D., Puccio, A., Rabinowitz, M., Robertson, C.S., Rosand, J., Sander, A., Satris, G., Schnyer, D., Seabury, S., Sherer, M., Taylor, S., Toga, A., Valadka, A., Vassar, M.J., Vespa, P., Wang, K., Yue, J.K., and Zafonte, R. (2019). Recovery after mild traumatic brain injury in patients presenting to US Level I Trauma Centers: a Transforming Research and Clinical Knowledge in Traumatic Brain Injury (TRACK-TBI) Study. JAMA Neurol. 76, 1049–1059. 31157856 10.1001/jamaneurol.2019.1313 PMC6547159

[6] Brooks, B.L., Kumari, J., and Virani, S. (2021). Family burden in adolescents with refractory postconcussion symptoms. J. Head Trauma Rehabil. doi: 10.1097/HTR.0000000000000717. 34320550

[7] Hyatt, K.S., Davis, L.L., and Barroso, J. (2015). Finding the new normal: accepting changes after combat-related mild traumatic brain injury. J. Nurs. Scholarsh. 47, 300–309. 25975680 10.1111/jnu.12143

[8] Silverberg, N.D., Panenka, W.J., and Iverson, G.L. (2018). Work productivity loss after mild traumatic brain injury. Arch. Phys. Med. Rehabil. 99, 250–256. 28760573 10.1016/j.apmr.2017.07.006

[9] Theadom, A., Barker-Collo, S., Jones, K., Kahan, M., Te Ao, B., McPherson, K., Starkey, N., and Feigin, V.; BIONIC4you Research Group. (2017). Work limitations 4 years after mild traumatic brain injury: a cohort study. Arch. Phys. Med. Rehabil. 98, 1560–1566. 28188778 10.1016/j.apmr.2017.01.010

[10] Young, J.T., and Hughes, N. (2020). Traumatic brain injury and homelessness: from prevalence to prevention. Lancet Public Health 5, e4–e5. 31806488 10.1016/S2468-2667(19)30225-7

[11] Ponsford, J., Willmott, C., Rothwell, A., Cameron, P., Kelly, A.M., Nelms, R., Curran, C., and Ng, K. (2000). Factors influencing outcome following mild traumatic brain injury in adults. J. Int. Neuropsychol. Soc. 6, 568–579. 10932476 10.1017/s1355617700655066

[12] Setnik, L., and Bazarian, J.J. (2007). The characteristics of patients who do not seek medical treatment for traumatic brain injury. Brain Inj. 21, 1–9. 17364514 10.1080/02699050601111419

[13] Victorian State Trauma Outcome Registry and Monitoring Group. (2022). Victorian State Trauma Registry. Victorian Government, Victorian Department of Health: Melbourne, Victoria, Australia.

[14] Feigin, V.L., Theadom, A., Barker-Collo, S., Starkey, N.J., McPherson, K., Kahan, M., Dowell, A., Brown, P., Parag, V., Kydd, R., Jones, K., Jones, A., and Ameratunga, S.;; BIONIC Study Group. (2013). Incidence of traumatic brain injury in New Zealand: a population-based study. Lancet Neurol. 12, 53–64. 23177532 10.1016/S1474-4422(12)70262-4

[15] Evans, J.A., van Wessem, K.J., McDougall, D., Lee, K.A., Lyons, T., and Balogh, Z.J. (2010). Epidemiology of traumatic deaths: comprehensive population-based assessment. World J. Surg. 34, 158–163. 19882185 10.1007/s00268-009-0266-1

[16] Myburgh, J.A., Cooper, D.J., Finfer, S.R., Venkatesh, B., Jones, D., Higgins, A., Bishop, N., and Higlett, T.; Australasian Traumatic Brain Injury Study Investigators for the ANZICS Clinical Trials Group. (2008). Epidemiology and 12-month outcomes from traumatic brain injury in Australia and New Zealand. J. Trauma 64, 854–862. 18404048 10.1097/TA.0b013e3180340e77

[17] Roquilly, A., Moyer, J.D., Huet, O., Lasocki, S., Cohen, B., Dahyot-Fizelier, C., Chalard, K., Seguin, P., Jeantrelle, C., Vermeersch, V., Gaillard, T., Cinotti, R., Demeure Dit Latte, D., Mahe, P.J., Vourc'h, M., Martin, F.P., Chopin, A., Lerebourg, C., Flet, L., Chiffoleau, A., Feuillet, F., and Asehnoune, K.; Atlanrea Study Group; Societe Francaise d'Anesthesie Reanimation Research Network. (2021). Effect of continuous infusion of hypertonic saline vs standard care on 6-month neurological outcomes in patients with traumatic brain injury: the COBI Randomized Clinical Trial. JAMA 325, 2056–2066. 34032829 10.1001/jama.2021.5561 PMC8150692

[18] Beck, B., Gantner, D., Cameron, P.A., Braaf, S., Saxena, M., Cooper, D.J., and Gabbe, B.J. (2018). Temporal trends in functional outcomes after severe traumatic brain injury: 2006–2015. J. Neurotrauma 35, 1021–1029. 29256832 10.1089/neu.2017.5287

[19] van Dijck, J., Bartels, R., Lavrijsen, J.C.M., Ribbers, G.M., Kompanje, E.J.O., and Peul, W.C.; all focus group participants. (2019). The patient with severe traumatic brain injury: clinical decision-making: the first 60 min and beyond. Curr. Opin. Crit. Care 25, 622–629. 31574013 10.1097/MCC.0000000000000671

[20] Robba, C., Graziano, F., Rebora, P., Elli, F., Giussani, C., Oddo, M., Meyfroidt, G., Helbok, R., Taccone, F.S., Prisco, L., Vincent, J.L., Suarez, J.I., Stocchetti, N., and Citerio, G.; SYNAPSE-ICU Investigators. (2021). Intracranial pressure monitoring in patients with acute brain injury in the intensive care unit (SYNAPSE-ICU): an international, prospective observational cohort study. Lancet Neurol. 20, 548–558. 34146513 10.1016/S1474-4422(21)00138-1

[21] Thelin, E., Al Nimer, F., Frostell, A., Zetterberg, H., Blennow, K., Nystrom, H., Svensson, M., Bellander, B.M., Piehl, F., and Nelson, D.W. (2019). A serum protein biomarker panel improves outcome prediction in human traumatic brain injury. J. Neurotrauma 36, 2850–2862. 31072225 10.1089/neu.2019.6375 PMC6761606

[22] Rubin, M.L., Yamal, J.M., Chan, W., and Robertson, C.S. (2019). Prognosis of six-month Glasgow Outcome Scale in severe traumatic brain injury using hospital admission characteristics, injury severity characteristics, and physiological monitoring during the first day post-injury. J. Neurotrauma 36, 2417–2422. 30860434 10.1089/neu.2018.6217 PMC6661910

[23] Wardlaw, C., Hicks, A.J., Sherer, M., and Ponsford, J.L. (2018). Psychological resilience is associated with participation outcomes following mild to severe traumatic brain injury. Front. Neurol. 9, 563. 30061858 10.3389/fneur.2018.00563 PMC6054998

[24] Steyerberg, E.W., Wiegers, E., Sewalt, C., Buki, A., Citerio, G., De Keyser, V., Ercole, A., Kunzmann, K., Lanyon, L., Lecky, F., Lingsma, H., Manley, G., Nelson, D., Peul, W., Stocchetti, N., von Steinbuchel, N., Vande Vyvere, T., Verheyden, J., Wilson, L., Maas, A.I.R., and Menon, D.K.; CENTER-TBI Participants and Investigators. (2019). Case-mix, care pathways, and outcomes in patients with traumatic brain injury in CENTER-TBI: a European prospective, multicentre, longitudinal, cohort study. Lancet Neurol. 18, 923–934. 31526754 10.1016/S1474-4422(19)30232-7

[25] Maas, A.I., Menon, D.K., Steyerberg, E.W., Citerio, G., Lecky, F., Manley, G.T., Hill, S., Legrand, V., and Sorgner, A.; CENTER-TBI Participants and Investigators. (2015). Collaborative European NeuroTrauma Effectiveness Research in Traumatic Brain Injury (CENTER-TBI): a prospective longitudinal observational study. Neurosurgery 76, 67–80. 25525693 10.1227/NEU.0000000000000575

[26] Yue, J.K., Vassar, M.J., Lingsma, H.F., Cooper, S.R., Okonkwo, D.O., Valadka, A.B., Gordon, W.A., Maas, A.I., Mukherjee, P., Yuh, E.L., Puccio, A.M., Schnyer, D.M., and Manley, G.T.; TRACK-TBI Investigators. (2013). Transforming research and clinical knowledge in traumatic brain injury pilot: multicenter implementation of the common data elements for traumatic brain injury. J. Neurotrauma 30, 1831–1844. 23815563 10.1089/neu.2013.2970 PMC3814815

[27] Yue, J.K., Yuh, E.L., Korley, F.K., Winkler, E.A., Sun, X., Puffer, R.C., Deng, H., Choy, W., Chandra, A., Taylor, S.R., Ferguson, A.R., Huie, J.R., Rabinowitz, M., Puccio, A.M., Mukherjee, P., Vassar, M.J., Wang, K.K.W., Diaz-Arrastia, R., Okonkwo, D.O., Jain, S., and Manley, G.T.; TRACK-TBI Investigators. (2019). Association between plasma GFAP concentrations and MRI abnormalities in patients with CT-negative traumatic brain injury in the TRACK-TBI cohort: a prospective multicentre study. Lancet Neurol. 18, 953–961.3145140910.1016/S1474-4422(19)30282-0

[28] Babl, F.E., Borland, M.L., Phillips, N., Kochar, A., Dalton, S., McCaskill, M., Cheek, J.A., Gilhotra, Y., Furyk, J., Neutze, J., Lyttle, M.D., Bressan, S., Donath, S., Molesworth, C., Jachno, K., Ward, B., Williams, A., Baylis, A., Crowe, L., Oakley, E., and Dalziel, S.R.; Paediatric Research in Emergency Departments International Collaborative. (2017). Accuracy of PECARN, CATCH, and CHALICE head injury decision rules in children: a prospective cohort study. Lancet 389, 2393–2402. 28410792 10.1016/S0140-6736(17)30555-X

[29] Katzenellenbogen, J.M., Atkins, E., Thompson, S.C., Hersh, D., Coffin, J., Flicker, L., Hayward, C., Ciccone, N., Woods, D., Greenland, M.E., McAllister, M., and Armstrong, E.M. (2018). Missing voices: profile, extent, and 12-month outcomes of nonfatal traumatic brain injury in Aboriginal and Non-Aboriginal adults in Western Australia using linked administrative records. J. Head Trauma Rehabil. 33, 412–423. 29601340 10.1097/HTR.0000000000000371

[30] Steyerberg, E.W., Mushkudiani, N., Perel, P., Butcher, I., Lu, J., McHugh, G.S., Murray, G.D., Marmarou, A., Roberts, I., Habbema, J.D., and Maas, A.I. (2008). Predicting outcome after traumatic brain injury: development and international validation of prognostic scores based on admission characteristics. PLoS Med. 5, e165. 18684008 10.1371/journal.pmed.0050165 PMC2494563

[31] Dijkland, S.A., Foks, K.A., Polinder, S., Dippel, D.W.J., Maas, A.I.R., Lingsma, H.F., and Steyerberg, E.W. (2020). Prognosis in moderate and severe traumatic brain injury: a systematic review of contemporary models and validation studies. J. Neurotrauma 37, 1–13. 31099301 10.1089/neu.2019.6401

[32] Maas, A.I., Murray, G.D., Roozenbeek, B., Lingsma, H.F., Butcher, I., McHugh, G.S., Weir, J., Lu, J., and Steyerberg, E.W.; IMPACT in TBI Study Group. (2013). Advancing care for traumatic brain injury: findings from the IMPACT studies and perspectives on future research. Lancet Neurol. 12, 1200–1210. 24139680 10.1016/S1474-4422(13)70234-5 PMC3895622

[33] Thelin, E.P., Nelson, D.W., Vehvilainen, J., Nystrom, H., Kivisaari, R., Siironen, J., Svensson, M., Skrifvars, M.B., Bellander, B.M., and Raj, R. (2017). Evaluation of novel computerized tomography scoring systems in human traumatic brain injury: an observational, multicenter study. PLoS Med. 14, e1002368. 28771476 10.1371/journal.pmed.1002368 PMC5542385

[34] Vande Vyvere, T., De La Rosa, E., Wilms, G., Nieboer, D., Steyerberg, E., Maas, A.I.R., Verheyden, J., van den Hauwe, L., and Parizel, P.M.; CENTER-TBI Participants and Investigators. (2020). Prognostic validation of the NINDS Common Data Elements for the radiologic reporting of acute traumatic brain injuries: a CENTER-TBI study. J. Neurotrauma 37, 1269–1282. 31813313 10.1089/neu.2019.6710

[35] Ponsford, J.L., Downing, M.G., Olver, J., Ponsford, M., Acher, R., Carty, M., and Spitz, G. (2014). Longitudinal follow-up of patients with traumatic brain injury: outcome at two, five, and ten years post-injury. J. Neurotrauma 31, 64–77. 23889321 10.1089/neu.2013.2997

[36] Ponsford, J., Downing, M., and Pechlivanidis, H. (2020). The impact of cultural background on outcome following traumatic brain injury. Neuropsychol. Rehabil. 30, 85–100. 29607708 10.1080/09602011.2018.1453367

[37] Ponsford, J.L., Spitz, G., and McKenzie, D. (2016). Using post-traumatic amnesia to predict outcome after traumatic brain injury. J. Neurotrauma 33, 997–1004. 26234939 10.1089/neu.2015.4025

[38] Spitz, G., Mahmooei, B.H., Ross, P., McKenzie, D., and Ponsford, J.L. (2019). Characterizing early and late return to work after traumatic brain injury. J. Neurotrauma 36, 2533–2540. 30924716 10.1089/neu.2018.5850

[39] O'Reilly, G.M., Curtis, K., Kim, Y., Rushworth, N., Mitra, B., Tee, J., Hunter, K., Ryder, C., Hendrie, D., and Fitzgerald, M.C. (2021). Establishing determinants and quality indicators for getting home alive following moderate to severe traumatic brain injury: the Australian Traumatic Brain Injury National Data Project. Emerg. Med. Australas. 33, 1121–1123. 34528396 10.1111/1742-6723.13861

[40] National Research Council (US) Committee on A Framework for Developing a New Taxonomy of Disease. (2011). Toward Precision Medicine: Building a Knowledge Network for Biomedical Research and a New Taxonomy of Disease. National Research Council: Washington, DC. 22536618

[41] Liu, N.T., Holcomb, J.B., Wade, C.E., Batchinsky, A.I., Cancio, L.C., Darrah, M.I., and Salinas, J. (2014). Development and validation of a machine learning algorithm and hybrid system to predict the need for life-saving interventions in trauma patients. Med. Biol. Eng. Comput. 52, 193–203. 24263362 10.1007/s11517-013-1130-x

[42] Evans, L.R., Fitzgerald, M.C., Varma, D., and Mitra, B. (2018). A novel approach to improving the interpretation of CT brain in trauma. Injury 49, 56–61. 28882376 10.1016/j.injury.2017.08.056

[43] Fitzgerald, M., Cameron, P., Mackenzie, C., Farrow, N., Scicluna, P., Gocentas, R., Bystrzycki, A., Lee, G., O'Reilly, G., Andrianopoulos, N., Dziukas, L., Cooper, D.J., Silvers, A., Mori, A., Murray, A., Smith, S., Xiao, Y., Stub, D., McDermott, F.T., and Rosenfeld, J.V. (2011). Trauma resuscitation errors and computer-assisted decision support. Arch. Surg. 146, 218–225. 21339436 10.1001/archsurg.2010.333

[44] Kreif, N., Grieve, R., Diaz, I., and Harrison, D. (2015). Evaluation of the effect of a continuous treatment: a machine learning approach with an application to treatment for traumatic brain injury. Health Econ. 24, 1213–1228. 26059721 10.1002/hec.3189 PMC4744663

[45] Slattery, P., Saeri, A.K., and Bragge, P. (2020). Research co-design in health: a rapid overview of reviews. Health Res. Policy Syst. 18, 17. 32046728 10.1186/s12961-020-0528-9 PMC7014755

[46] Australian Research Data Commons. (2021). FAIR data. Making Australia's research data FAIR (findable, accessible, interoperable and reusable) supports knowledge discovery and innovation. Monash University: Caulfield East, VIC, Australia.

[47] Vos, T., Barker, B., Begg, S., Stanley, L., and Lopez, A.D. (2009). Burden of disease and injury in Aboriginal and Torres Strait Islander Peoples: the Indigenous health gap. Int. J. Epidemiol. 38, 470–477. 19047078 10.1093/ije/dyn240

[48] Randall, S.M., Brown, A.P., Ferrante, A.M., and Boyd, J.H. (2019). Privacy preserving linkage using multiple match-keys. Int. J. Popul. Data Sci 4, 1094. 32935028 10.23889/ijpds.v4i1.1094 PMC7482515

[49] Azzam, N., Oei, J.L., Adams, S., Bajuk, B., Hilder, L., Mohamed, A.L., Wright, I.M.R., and Holland, A.J.A. (2018). Influence of early childhood burns on school performance: an Australian population study. Arch. Dis. Child. 103, 444–451. 29187346 10.1136/archdischild-2017-313355

