# Supplementary material for: AUS-TBI: The Australian Health Informatics Approach to Predict Outcomes and Monitor Intervention Efficacy after Moderate-to-Severe Traumatic Brain Injury
Source: Neurotrauma Rep. 2022 Jun 7;3(1):217–23. doi: 10.1089/neur.2022.0002 (PMC9279124; doi:10.1089/neur.2022.0002)
Supplement: Supplemental data [file Suppl_Data.docx]

The AUS-TBI Investigators

Tara Alexander bw Australasian Rehabilitation Outcomes Centre (AROC), Faculty of Business and Law,

0000-0001-5234-7821 University of Wollongong, Wollongong, NSW Australia

bx Australian Health Services Research Institute (AHSRI), Faculty of Business and Law,

University of Wollongong, Wollongong, NSW Australia

Vicki Anderson bu Psychology Service, The Royal Children's Hospital, Melbourne, VIC Australia

bv Clinical Sciences Research, Murdoch Children’s Research Institute, Melbourne, VIC Australia

Ana Antonic-Baker k Department of Neuroscience, Central Clinical School, Monash University, Melbourne,

Australia

Elizabeth Armstrong cx School of Medical and Health Sciences, Edith Cowan University, Perth, WA Australia

Franz E Babl db Department of Emergency Medicine, The Royal Children’s Hospital, Melbourne, Australia

bg Departments of Paediatrics and Critical Care, University of Melbourne, Melbourne, Australia

dc Murdoch Children’s Research Institute, Melbourne, VIC Australia

Matthew K Bagg a Curtin Health Innovation Research Institute, Faculty of Health Sciences, Curtin University,

0000-0002-4812-3814 Bentley, WA Australia

b Perron Institute for Neurological and Translational Science, Nedlands, WA Australia

c Centre for Pain IMPACT, Neuroscience Research Australia, Sydney, NSW Australia

d WA SportsMed Physiotherapy, Perth, WA Australia

Zsolt J Balogh ax Department of Traumatology, John Hunter Hospital and University of Newcastle, Newcastle,

NSW Australia

Karen M Barlow bp Acquired Brain Injury in Children Research Program, Queensland Children's Hospital,

Brisbane, QLD Australia

bq Centre for Children's Health Research, University of Queensland, Brisbane, QLD Australia

Judith Bellapart di Department of Intensive Care Services, Royal Brisbane and Women's Hospital, Brisbane,

QLD Australia

dj Faculty of Medicine, University of Queensland, Brisbane, QLD Australia

Niranjan Bidargaddi o Flinders Digital Health Centre, College of Medicine & Public Health, Flinders University,

Adelaide, Australia

Erika Bosio aw Centre for Clinical Research in Emergency Medicine, Harry Perkins Institute of Medical

Research, Nedlands, WA Australia

ay School of Biomedical Science, University of Western Australia, Crawley, WA Australia

az School of Medicine, University of Western Australia, Crawley, WA Australia

Peter Bragge v BehaviourWorks Australia, Monash Sustainable Development Institute, Monash University,

Melbourne, Australia

Michael Bynevelt do School of Surgery, The University of Western Australia, Crawley, WA Australia

dp Neurological Intervention and Imaging Service of Western Australia, Sir Charles Gairdner

Hospital, Nedlands, WA Australia

Karen Caeyenberghs dk Mary MacKillop Institute for Health Research, Australian Catholic University, Melbourne, VIC

Australia

Peter A Cameron ca National Trauma Research Institute, Melbourne, VIC Australia

e School of Public Health and Preventive Medicine, Monash University, Melbourne, Australia

de Emergency and Trauma Centre, The Alfred Hospital, Melbourne, VIC Australia

Jacquelin Capell bw Australasian Rehabilitation Outcomes Centre (AROC), Faculty of Business and Law,

0000-0003-1054-9155 University of Wollongong, Wollongong, NSW Australia

bx Australian Health Services Research Institute (AHSRI), Faculty of Business and Law,

University of Wollongong, Wollongong, NSW Australia

Kevin E K Chai ab School of Population Health, Faculty of Health Sciences, Curtin University, Bentley, WA

Australia

cw Curtin Institute for Computation, Curtin University, Bentley, WA Australia

Lyndsey E Collins-Praino w School of Biomedicine, University of Adelaide, Adelaide, SA Australia

D J Jamie Cooper bn Australian and New Zealand Intensive Care Research Centre, School of Public Health and

Preventive Medicine, Monash University, Melbourne, VIC Australia

bo Department of Intensive Care and Hyperbaric Medicine, The Alfred, Melbourne, VIC

Australia

Gill Cowen n School of Medicine, Faculty of Health Sciences, Curtin University, Bentley, WA Australia

Louise M Crowe bv Clinical Sciences Research, Murdoch Children’s Research Institute, Melbourne, VIC Australia

bg Department of Paediatrics, University of Melbourne, Melbourne, Australia

Jennifer Cullen bp Synapse, Brisbane, QLD Australia

bq James Cook University, Townsville, QLD Australia

br Menzies Health Institute Queensland, Griffith University, Brisbane, QLD Australia

Kate Curtis ac Susan Wakil School of Nursing and Midwifery, Faculty of Medicine and Health, The

0000-0002-3746-0348 University of Sydney, NSW, Australia

ad Illawarra Shoalhaven LHD, Wollongong, NSW Australia

ae Illawarra Health and Medical Research Institute, Wollongong, NSW Australia

af George Institute for Global Health, Newtown, NSW Australia

Anthony Delaney al Division of Critical Care, The George Institute for Global Health, Newtown, NSW Australia

am Malcolm Fisher Department of Intensive Care Medicine, Royal North Shore Hospital, Sydney,

NSW Australia

cr Northern Clinical School, Sydney Medical School, Sydney University, Sydney, NSW Australia

bn Australian and New Zealand Intensive Care Research Centre, School of Public Health and

Preventive Medicine, Monash University, Melbourne, VIC Australia

Sandra Eades n School of Medicine, Faculty of Health Sciences, Curtin University, Bentley, WA Australia

Gary F Egan cm Monash Biomedical Imaging, Monash University, Melbourne, VIC Australia

cn School of Psychological Sciences, Monash University, Melbourne, VIC Australia

Mark S Elcock dz Discipline of Public Health and Tropical Medicine, College of Public Health, Medical and Veterinary Sciences, James Cook University, Townsville, QLD Australia

ae Prevention Division, Department of Health, Aeromedical Retrieval and Disaster Management Branch, Brisbane, QLD Australia

Daniel Y Ellis x Department of Trauma, Royal Adelaide Hospital, Adelaide, SA Australia

y Statewide South Australian Trauma Service, SA Australia

z School of Public Health and Tropical Medicine, James Cook University, QLD Australia

Ari Ercole at Division of Anaesthesia, University of Cambridge, Addenbrooke’s Hospital, Cambridge UK

au Cambridge Centre for AI in Medicine, University of Cambridge, UK

Daniel M Fatovich av Emergency Medicine, Royal Perth Hospital, University of Western Australia, Perth, WA

Australia

aw Centre for Clinical Research in Emergency Medicine, Harry Perkins Institute of Medical

Research, Nedlands, WA Australia

Murray J Fisher ac Susan Wakil School of Nursing and Midwifery, Faculty of Medicine and Health, The

University of Sydney, NSW, Australia

cv Royal Rehab, Ryde, Sydney, NSW Australia

Mark Fitzgerald ca National Trauma Research Institute, Melbourne, VIC Australia

Melinda Fitzgerald a Curtin Health Innovation Research Institute, Faculty of Health Sciences, Curtin University,

Bentley, WA Australia

b Perron Institute for Neurological and Translational Science, Nedlands, WA Australia

Jennifer Fleming cj School of Health and Rehabilitation Sciences, The University of Queensland, Brisbane, QLD

Australia

Roslyn Francis dv Department of Health, Government of Western Australia, Perth, WA Australia

Belinda Gabbe e School of Public Health and Preventive Medicine, Monash University, Melbourne, Australia

f Health Data Research UK, Swansea University Medical School, Swansea University, Singleton

Park, United Kingdom

Adelle Gadowski e School of Public Health and Preventive Medicine, Monash University, Melbourne, Australia

Timothy J Geraghty p Division of Rehabilitation, Princess Alexandra Hospital, Brisbane, QLD Australia

q The Hopkins Centre, Menzies Health Institute Queensland, Griffith University, Brisbane, QLD

Australia

John Gilroy ar Aboriginal and Torres Strait Islander Research, Faculty of Medicine and Health, The

University of Sydney, Sydney, NSW Australia

Mitchell A Hansen ck Department of Neurosurgery, John Hunter Hospitals and University of Newcastle,

Newcastle, NSW Australia

James E Harrison df College of Medicine and Public Health, Flinders University, Bedford Park, SA Australia

Luke J Haseler a Curtin Health Innovation Research Institute, Faculty of Health Sciences, Curtin University,

Bentley, WA Australia

Leanne Hassett ag Institute for Musculoskeletal Health, The University of Sydney/Sydney Local Health District,

Sydney, Australia

ah Sydney School of Health Sciences, Faculty of Medicine and Health, The University of Sydney,

Sydney, Australia

Sarah C Hellewell a Curtin Health Innovation Research Institute, Faculty of Health Sciences, Curtin University,

Bentley, WA Australia

n School of Medicine, Faculty of Health Sciences, Curtin University, Bentley, Western

Australia, Australia

b Perron Institute for Neurological and Translational Science, Nedlands, WA Australia

Amelia J Hicks g Turner Institute for Brain and Mental Health, School of Psychological Sciences, Monash

University, Melbourne, Australia

h Monash Epworth Rehabilitation Research Centre, Epworth Healthcare, Melbourne, Australia

Andrew F Hill bh College of Science, Health and Engineering, La Trobe University, Bundoora, VIC Australia

Andrew J A Holland cf The Children's Hospital at Westmead Clinical School, Faculty of Medicine and Health, The

0000-0003-3745-8704 University of Sydney, Westmead, NSW Australia

Stephen Honeybul cy Department of Neurosurgery, Sir Charles Gairdner Hospital, Nedlands, WA Australia

cz Department of Neurosurgery, Royal Perth Hospital, Perth, WA Australia

Rosalind L Jeffree cg Kenneth G. Jamieson Department of Neurosurgery, Royal Brisbane and Women’s Hospital,

Brisbane, QLD Australia

ch School of Medicine – Royal Brisbane Clinical School, University of Queensland, Brisbane,

QLD Australia

Chris Joyce ct Intensive Care Unit, Princess Alexandra Hospital, Brisbane, QLD Australia

cu School of Medicine, University of Queensland, Brisbane, QLD Australia

Elizabeth Kendall br Menzies Health Institute Queensland, Griffith University, Brisbane, QLD Australia

Kate King ax Department of Traumatology, John Hunter Hospital and University of Newcastle, Newcastle, NSW Australia

Natasha A Lannin k Department of Neuroscience, Central Clinical School, Monash University, Melbourne,

Australia

j Alfred Health, Melbourne, Australia

Meng Law dm Alzheimer's Disease Research Center, Keck School of Medicine, University of Southern

California, Los Angeles, CA, USA

dn Department of Neurological Surgery, Keck School of Medicine, University of Southern

California, Los Angeles, CA, USA

do Department of Neuroscience and Radiology, Monash University, Alfred Health, Melbourne,

VIC Australia

Andrew I R Maas bc Department of Neurosurgery, Antwerp University Hospital and University of Antwerp,

Edegem, Belgium

Adam Mahoney bi Trauma Service, Royal Hobart Hospital, Hobart, TAS Australia

bj 2nd General Health Battalion, Australian Defence Force

Skye McDonald ds School of Psychology, University of New South Wales, Sydney, NSW Australia

Stuart J McDonald k Department of Neuroscience, Central Clinical School, Monash University, Melbourne,

Australia

Ancelin McKimmie e School of Public Health and Preventive Medicine, Monash University, Melbourne, Australia

Robert McNamara bc Department of Intensive Care Medicine, Royal Perth Hospital, Perth, WA Australia

n School of Medicine, Faculty of Health Sciences, Curtin University, Bentley, Western

Australia, Australia

Shiv Meka dv Department of Health, Government of Western Australia, Perth, WA Australia

David K Menon at Division of Anaesthesia, University of Cambridge, Addenbrooke’s Hospital, Cambridge UK

da Wolfson Brain Imaging Centre, University of Cambridge, Cambridge, UK

Gary Mitchell an Emergency and Trauma Unit, Royal Brisbane and Women's Hospital, Brisbane, QLD Australia

ao Royal Brisbane Clinical Unit, University of Queensland, Brisbane, QLD Australia

ap Jamieson Trauma Institute, Brisbane, QLD Australia

cp Queensland Rugby Union, Brisbane, QLD Australia

Rowena Mobbs dq Brain & Mind Centre, University of Sydney, Camperdown, NSW Australia

dr Macquarie University, Sydney, NSW Australia

Fatima A Nasrallah ci Queensland Brain Institute, The University of Queensland, Brisbane, QLD Australia

Virginia Newcombe at Division of Anaesthesia, University of Cambridge, Addenbrooke’s Hospital, Cambridge UK

Terence J O'Brien k Department of Neuroscience, Central Clinical School, Monash University, Melbourne,

Australia

John H Olver l Epworth Healthcare, Melbourne, Australia

m Department of Medicine, Monash University, Melbourne, Australia

Gerard M O'Reilly ca National Trauma Research Institute, Melbourne, VIC Australia

de Emergency and Trauma Centre, The Alfred Hospital, Melbourne, VIC Australia

e School of Public Health and Preventive Medicine, Monash University, Melbourne, Australia

Tamara Ownsworth q The Hopkins Centre, Menzies Health Institute Queensland, Griffith University, Brisbane, QLD

Australia

as School of Applied Psychology, Griffith University, Brisbane, QLD Australia

Paul M Parizel du University of Antwerp, Edegem, Belgium

dx Department of Radiology, Royal Perth Hospital & University of Western Australia, Perth, WA Australia

dy West Australian National Imaging Facility Node, Nedlands, WA Australia

Michael Parr aj Intensive Care Unit, Liverpool Hospital, University of New South Wales, Sydney, NSW

Australia

ak Intensive Care Unit, Macquarie University Hospital,

Macquarie University, Sydney, NSW Australia

Jennie L Ponsford g Turner Institute for Brain and Mental Health, School of Psychological Sciences, Monash

University, Melbourne, Australia

h Monash Epworth Rehabilitation Research Centre, Epworth Healthcare, Melbourne, Australia

Michael C Reade bs Faculty of Medicine, University of Queensland, Royal Brisbane and Women's Hospital,

Brisbane, QLD Australia

bt Joint Health Command, Australian Defence Force, Canberra, ACT Australia

Sandy Reeder k Department of Neuroscience, Central Clinical School, Monash University, Melbourne,

Australia

aq Department of Epidemiology and Preventive Medicine, School of Public Health and

Preventive Medicine, Monash University, Melbourne, Australia

Christopher Reid eb School of Public Health, Faculty of Health Sciences, Curtin University, Perth, WA Australia

aq Department of Epidemiology and Preventive Medicine, School of Public Health and Preventive Medicine, Monash University, Melbourne, Australia

Suzanne Robinson ab School of Population Health, Faculty of Health Sciences, Curtin University, Bentley, WA

Australia

Stephen E Rose dt The Australian e-Health Research Centre, CSIRO, Brisbane, QLD Australia

Jeffrey V Rosenfeld cl Department of Neurosurgery, The Alfred, Melbourne, VIC Australia

bz Department of Surgery, Monash University, Melbourne, VIC Australia

cl F. Edward Hébert School of Medicine, Uniformed Services University of The Health Sciences,

Bethesda, MD, USA

Jason P Ross bm Molecular Diagnostic Solutions, Health and Biosecurity, CSIRO, Australia

Nick Rushworth r Brain Injury Australia, Sydney, NSW Australia

Adam Scheinberg ai Neurodevelopment and Rehabilitation Research, Murdoch Children's Research Institute,

Parkville, VIC Australia

bg Department of Paediatrics, University of Melbourne, Melbourne, Australia

Bridgette D Semple k Department of Neuroscience, Central Clinical School, Monash University, Melbourne,

Australia

j Alfred Health, Melbourne, Australia

aa Department of Medicine (Royal Melbourne Hospital), The University of Melbourne, Parkville,

VIC Australia

Sandy R Shultz k Department of Neuroscience, Central Clinical School, Monash University, Melbourne,

Australia

Grahame K Simpson ba Brain Injury Rehabilitation Research Group, Ingham Institute for Applied Medical Research,

Sydney, NSW Australia

bb John Walsh Centre for Rehabilitation Research, Sydney School of Medicine, University of

Sydney, Sydney, NSW Australia

Warwick J Teague bd Trauma Service, The Royal Children’s Hospital, Melbourne, Australia

0000-0003-4747-6025

be Department of Paediatric Surgery, The Royal Children’s Hospital, Melbourne, Australia

bf Surgical Research, Murdoch Children’s Research Institute, Melbourne, Australia

bg Department of Paediatrics, University of Melbourne, Melbourne, Australia

Leanne Togher cq Speech Pathology, School of Health Sciences, Faculty of Medicine and Health, The University

of Sydney, Sydney, Australia

Andrew A Udy bn Australian and New Zealand Intensive Care Research Centre, School of Public Health and

Preventive Medicine, Monash University, Melbourne, VIC Australia

bo Department of Intensive Care and Hyperbaric Medicine, The Alfred, Melbourne, VIC

Australia

Kirsten Vallmuur dl Centre for Healthcare Transformation, Australian Centre for Health Services Innovation,

Queensland University of Technology, Brisbane, QLD Australia

ap Jamieson Trauma Institute, Brisbane, QLD Australia

Dinesh Varma by Department of Radiology, The Alfred Hospital, Melbourne, VIC Australia

bz Department of Surgery, Monash University, Melbourne, VIC Australia

ca National Trauma Research Institute, Melbourne, VIC Australia

James Vickers dg Wicking Dementia Research and Education Centre, College of Health and Medicine,

University of Tasmania, Hobart, TAS Australia

Janet Wagland bs Brightwater Group, Osborne Park, WA Australia

James Walsham ct Intensive Care Unit, Princess Alexandra Hospital, Brisbane, QLD Australia

cu School of Medicine, University of Queensland, Brisbane, QLD Australia

Adam J Wells s Department of Neurosurgery, Adelaide Hospital, Adelaide, SA Australia

t Department of Surgery, University of Adelaide, Adelaide, SA Australia

u Neurosurgical Research Foundation, Adelaide, SA Australia

Luke Whiley co Health Futures Institute, Murdoch University, Perth, WA Australia

b Perron Institute for Neurological and Translational Science, Nedlands, WA Australia

Gavin Williams bk Department of Physiotherapy, Epworth Healthcare, Melbourne, VIC Australia

bl Department of Physiotherapy, University of Melbourne, Melbourne, VIC, Australia

Jodie K Williams dh National Critical Care and Trauma Response Centre, Royal Darwin Hospital, Darwin, NT

Australia

David K Wright k Department of Neuroscience, Central Clinical School, Monash University, Melbourne, Australia

Jesse T Young cb Centre for Health Equity, Melbourne School of Population and Global Health, The University

0000-0001-5702-372X of Melbourne, Parkville, VIC Australia

cc Centre for Adolescent Health, Murdoch Children’s Research Institute, Parkville, VIC Australia

cd School of Population and Global Health, The University of Western Australia, Perth, WA

Australia

ce National Drug Research Institute, Curtin University, Perth, WA Australia

Heidi Zeeman q The Hopkins Centre, Menzies Health Institute Queensland, Griffith University, Brisbane, QLD

Australia
